# The Nexus between Urbanization and Traffic Accessibility in the Middle Reaches of the Yangtze River Urban Agglomerations, China

**DOI:** 10.3390/ijerph18073828

**Published:** 2021-04-06

**Authors:** Fengjian Ge, Wanxu Chen, Yuanyuan Zeng, Jiangfeng Li

**Affiliations:** 1 Department of Land Resource Management, School of Public Administration, China University of Geosciences, Wuhan 430074, China; fjge@cug.edu.cn (F.G.); jfli@cug.edu.cn (J.L.); 2 Department of Geography, School of Geography and Information Engineering, China University of Geosciences, No. 388 Lumo Road, Wuhan 430074, China; 3 4th Planning & Design Office, Guangdong Urban & Rural Planning and Design Institute, No. 483 Nanzhou Road, Haizhu District, Guangzhou 510290, China

**Keywords:** urbanization, traffic accessibility, bivariate spatial autocorrelation analysis, spatial regression, the Middle Reaches of the Yangtze River Urban Agglomerations, China

## Abstract

China has entered the stage where urban agglomerations underpin and spearhead the county’s urbanization. Urban agglomerations in China have become economic growth poles, and the constantly improving transport networks in these agglomerations bring about opportunities for redistributing labor forces and promoting regional economic development, trade, and social progress for all. This is the foundation and fuel for urban development. However, lack of knowledge of the spatial features of, and the interrelationship between, regional urbanization and traffic accessibility constrains effective urban planning and decision-making. To fill this gap, this study attempted to evaluate the spatiotemporal distribution characteristics of urbanization levels and traffic accessibility in 1995, 2005, and 2015 in the Middle Reaches of the Yangtze River Urban Agglomerations (MRYRUA), China. The spatial interaction, spatial dependence effect, and spatial spillover effect between urbanization and traffic accessibility were tested by employing the bivariate spatial autocorrelation model and spatial regression models. The results showed that the urbanization level and traffic accessibility in the MRYRUA shot up over time and manifested similar spatial distribution characteristics. The global bivariate spatial autocorrelation coefficients were positive and significant during the period studied, and the main relationship types were the high urbanization and high traffic accessibility types and low urbanization and low traffic accessibility types. The spatial regression results showed that there was a significant positive association between urbanization and traffic accessibility, but with a significant scale effect. Urbanization is not only affected by the traffic accessibility of the individual grid unit but also by those in the adjacent or further grid units. The findings in this study provide important implications for urbanization development and transportation planning. The spatial dependence effect and spatial spillover effect between urbanization and traffic accessibility should be considered in future urban planning and transportation planning. The rational allocation of resources and inter-regional joint management can be an effective path toward regional sustainability.

## 1. Introduction

Over the past 40 years of reform and opening-up, China has made great progress in terms of urbanization. The urban population in China in 1978 was only 17.9%, while it reached 56.1% in 2015 [1]. Urban agglomerations have become the main platforms for promoting new-type urbanization in China. With the increase of cross-regional exchanges, cooperation, and flow of production factors, transportation facilities have played an increasingly prominent role in delivering efficient growth of urbanization, especially in urban agglomerations. However, the previous literature has lacked studies of the spatial relationship between urbanization and traffic accessibility. Clearly identifying the impact of traffic accessibility on urbanization is of great importance for the formulation of relevant planning and policies for rapid urbanization.

Empirical studies have evidenced that urbanization is, directly or indirectly, related to the widespread apprehension of modern society regarding resources, the environment, and sustainable development; concerns include human health [2], environmental pollution [3,4], energy consumption [5], carbon emissions [6], and global climate change [7,8]. A comprehensive evaluation of the spatiotemporal evolution features of urbanization and exploration of the key influencing factors of urbanization is necessary for sustainable urban development. This is also the theoretical premise for comprehensively deepening the development of urbanization in China as well as the basic support for recognizing the negative impact of urbanization and making suggestions for related compensation or elimination.

Since 1978, world-class traffic networks have been emerging in China, facilitating population mobility, economic development, and trade links domestically and internationally. Currently, China′s urbanization is entering a phase of high-quality development, and the central and local governments are grappling with new challenges. To meet the expectation of ‘corner overtaking’, the urgent need for inter-regional coordinated development has been increasingly reflected almost everywhere in recent years. To that end, the government has carried out top-level design and put forward the development concept of urban agglomerations. Hence, it is imperative to promote regional integrated development by using transport construction as a lever [9]. As an embodiment of regional integrated development, urban agglomerations have become a new spatial unit of competitive edge in the ever-increasing globalization and world economic competition. At present, the Chinese government capitalizes on the construction of urban agglomerations as a policy tool to have a favorable position in global and regional competition. Existing studies have focused on the spatiotemporal development patterns of urbanization, the potential hidden dangers, and the relevant governance suggestions, but have rarely accurately identified the determinant factors of urbanization development. To some extent, this limits the practicability of the corresponding policy recommendations [10]. Urban agglomerations are the highest level of spatial organization in the mature stage of urban development and have gradually become the main morphology of new-type urbanization and an important embodiment of the modernization drive. The effort in exploring the spatial relationship between urbanization and traffic networks in urban agglomerations can provide scientific reference for both proper formulation of urbanization planning and the integrated internal development of urban agglomerations.

Urbanization is often combined with population migration [11], economic development [12,13], developed land expansion [14,15], and social welfare improvement [16]. Population migration is the spatial redistribution of labor forces under certain conditions, and it generally encompasses population mobility between urban and rural areas or across different regions [17,18]. No matter what factors contribute to population migration, two preconditions must be considered: the unbalanced development of inflow and outflow areas (e.g., economy, living standards) [19], and regional connectivity (e.g., transportation) [20]. The imbalance of regional development has stimulated self-motivated migration; the development of transportation addresses the urgent demands associated with this movement. Developed areas have become the top-of-mind choice of migrants within a certain region due to the attractive economic conditions and living standards. In addition, superior transportation conditions have also enhanced the competitiveness and convenience of urban agglomerations, thus appealing to immigrants.

Additionally, economic development is one of the main purposes of urbanization in developing countries and is usually closely correlated to the continuous improvement of traffic networks [21,22]. The location of traffic networks present differentiated regional growth opportunities; however, requirements for transport conditions vary greatly. Specifically, traffic networks augment not only the industrial sector through the facilitation of the input and output of production factors but also tertiary industries by raising population density and consumption power to a certain level. As the carrier of the flow of production factors, traffic networks bridge production with consumption, regional supply with demand, which can attract, multiply, and synthesize industry, capital, and population. The development and improvement of traffic networks can improve the regional delivery capacity and sharpen its comparative competitive edge [23]. The scale economy and combined effect create conditions for the utilization of this advantage. In short, the traffic networks meet the requirements of comparative advantage in product exportation.

City aggrandizement, which leverages traffic network extension, has provided impetus for land urbanization [24]. To strengthen the communication and cooperation within the integrated regions and to consolidate the competitiveness of the urban agglomerations at home and even abroad, decision makers consider traffic construction as a spearhead, striving to break down the regional spatial barriers and promote integration; examples of this include the Wuhan and Zhejiang city circle intercity railways. Travel in the daily life of urban residents and economic activities depend on the connection of urban traffic networks and transport convenience. This has also become an imperative driver in making housing purchase decisions [25]. Transport planning effectively raises the socioeconomic value of the land along its network, which is widely appreciated by developers, especially in the context of the growing scarcity of available land for development in mature cities. Empirical research has proven that there is a significant positive spatial correlation between transport location and construction land expansion, especially in urban agglomerations [26,27]. In addition, quality-oriented urbanization drivers usually include the promotion of social welfare, such as pensions, medical services, and other forms of social security. Due to the dual household registration system in urban and rural areas, the rural population migrating to cities could not enjoy the same level of social welfare as urban dwellers and would be confronted with barriers in applying for household registration in cities, such as education requirements. When considering the compound definition of urbanization in light of transportation, transport construction helps raise urbanization levels by promoting population migration, economic development, urban expansion, and social welfare improvement.

In this study, the Middle Reaches of the Yangtze River urban agglomerations (MRYRUA) were selected as the study case to explore the spatiotemporal evolution of urbanization and traffic networks and their spatial interaction in 1995, 2005, and 2015. The propose of this study is to provide a scientific reference for other similar urban agglomerations in China and other developing countries to promote urbanization through the improvement of traffic networks. The MRYRUA has great transportation advantages. Transportation is facilitated by developed river systems, including the Yangtze River, the Han River, the Yuanjiang River, the Xiangjiang River, and the Ganjiang River. In addition, land transportation and shipping traffic in the MRYRUA are developing rapidly. The Beijing-Guangzhou Line, Beijing-Kowloon Line, Jiaoliu Line, Zhejiang-Jiangxi Line, and other major railway lines connect the whole country. There are also more than 300 air routes in the MRYRUA connecting major cities and countries around the world.

To this end, this study makes progress in three aspects: (1) scoping the MRYRUA, a national urban agglomeration, as a case study to better provide scientific guidance and reference for cross-regional collaborative governance in similar urban agglomerations in the world; (2) examining the spatiotemporal evolution features of urbanization and traffic accessibility in the MRYRUA at 5 × 5 km^2^ and 10 × 10 km^2^ grid scales; (3) investigating the spatial interaction and spatial dependence effect of urbanization and traffic accessibility in the MRYRUA with a set of spatial regression models.

The rest of this study is arranged as follows. In Section 2, the introduction of the study area, the data used for empirical research, and the methods employed in this study are elaborated. In Section 3, the spatiotemporal patterns of traffic accessibility and urbanization and their spatial interactions are analyzed for the MRYRUA for 1995, 2005, and 2015. Section 4 sheds light on the collaborative relationship between the development of urbanization and traffic accessibility, on the implications of relevant urban planning, and on future research directions. Section 5 includes a brief summary.

## 2. Materials and Methods

### 2.1. Study Area

**Figure 1:**
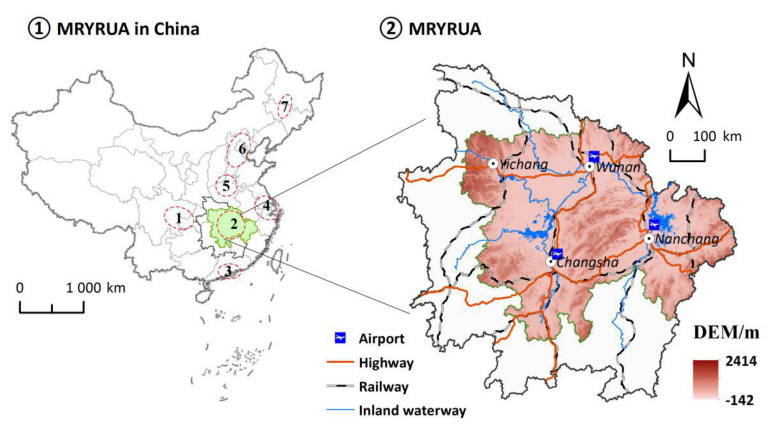
Location of the Middle Reaches of the Yangtze River Urban Agglomerations (MRYRUA) in China. Notes: 1, 2, 3, 4, 5, 6, and 7 in the figure represent Chengdu-Chongqing Urban Agglomeration, the Middle Reaches of the Yangtze River Urban Agglomerations, Pearl River Delta Urban Agglomeration, Yangtze River Delta Urban Agglomeration, Central Plains Urban Agglomeration, Beijing-Tianjin-Hebei Urban Agglomeration, and Harbin-Changchun Urban Agglomeration, respectively.

As one of the seven national-level urban agglomerations approved by the State Council, the MRYRUA is located at the center of the other four urban agglomerations (Yangtze River Delta Urban Agglomerations, Pearl River Delta Urban Agglomerations, Chengdu-Chongqing Urban Agglomerations, and Central Plains Urban Agglomerations). The MRYRUA composed of Wuhan Megalopolis, Changsha–Zhuzhou–Xiangtan Urban Agglomerations, Jingmen–Jingzhou–Yichang Urban Agglomerations, and Poyang Lake Urban Agglomerations (26°07′05″ N–30°23′ N,110°15′ E–118°28′58″ E) (Figure 1). As the pioneer of new-type urbanization in central and western China, the inland open-up pilot cooperation zone, and the pioneer of ‘two types society’ construction (resource conserving and environmentally friendly), the MRYRUA is in a leading position in China with regard to the new-type urbanization, transportation, and other aspects. In addition, with the implementation of the Yangtze River Economic Belt Strategy, the Rise of Central China Plan, and the Triangle of Central China, the MRYRUA has become a new growth pole of China′s economic development. The special geographic location and strategic development plan highlight the role of the MRYRUA as an important transportation and economic hub. The fully fledged three-dimensional transport network of water, land, and air has become an important driver in promoting the flow of population and capital in the MRYRUA, making it a new engine for accelerating urbanization. In this light, it is of great significance to explore the spatial relationship between traffic accessibility and urbanization levels in the MRYRUA, with the propose of strengthening the transport construction and realizing the new-type urbanization blueprint in a rapid manner.

### 2.2. Data Sources and Processing

The adopted water, land, and air transport data of 1995, 2005, and 2015 in the MRYRUA were sourced from the historical transport maps of Hunan, Hubei, and Jiangxi in the corresponding years. The 1 × 1 km^2^ resolution population and GDP raster data in 1995, 2005, and 2015 refer to the Resource and Environment Science Data Center (RESDC) of the Chinese Academy of Sciences (http://www.resdc.cn, accessed on 1 January 2021). The proportion of developed land was adopted from Landsat TM/ETM remote sensing image interpretation data in 1995, 2005, and 2015, with a spatial resolution of 30 × 30 m^2^. Digital elevation model data were adopted from the geospatial data cloud (http://www.gscloud.cn/, accessed on 1 January 2021).

### 2.3. Methods

#### 2.3.1. Urbanization Measurement

Urbanization is a complex system, encompassing four main sub-systems (e.g., population urbanization, economic urbanization, social urbanization, and land urbanization). In such a system, any trade-off can comprise the overall benefits of urbanization [28]. Population urbanization is the core, economic urbanization is the driving force, and land urbanization is the guarantee [28]. Population urbanization and economic urbanization lay a solid foundation for urbanization. Social urbanization and land urbanization are manifested in the society and space, respectively [29]. As it is difficult to have access to socioeconomic data related to social urbanization, it will not be discussed in this study. Referring to the research results of Chen et al. [30], in this study, population density (PD), economic density (GDPD), and the percentage of developed land (DLP) were selected to quantify population urbanization, economic urbanization, and land urbanization, respectively. The indicators of PD and GDPD were extracted by Arc Toolbox/Spatial Analyst Tools/Zonal/Zonal Statistics of ArcGIS10.3 with the 1×1 km^2^ resolution population, and GDP raster data from 1995, 2005, and 2015 refer to the Resource and Environment Science Data Center (RESDC) of the Chinese Academy of Sciences (http://www.resdc.cn, accessed on 1 January 2021). DLP is extracted from the land use data from 1995, 2005, and 2015, with a spatial resolution of 30 × 30 m^2^.

Furthermore, there are different development stages of urbanization, and the population, economic growth rate, and land expansion degree of each stage are not the same as in other stages. Even at the same time point, different regions are at different stages, and their performance is very different. Taking this into consideration, we used the equal weight coefficient to synthesize three dimensions to reflect the level of regional urbanization (UL). To eliminate the dimensional inconsistencies, the deviation standardization was used to standardize these three indicators (Equation (1)). The equation is as follows:

(1) $$\bar{X}=\frac{X-X_{\min}}{X_{\max}-X_{\min}}$$

(2) $$UL=\left(PD+GDPD+DLP\right)/3$$

where, $\bar{X}$ denotes the standardized urbanization indicator and *X*, *X_min_*, and *X_max_* are respectively the urbanization indicator, minimum value of urbanization indicator and maximum value of urbanization indicator. *UL* denotes the urbanization level. *PD, GDPD*, and *DLP* are respectively the population density, economic density, and the percentage of developed land.

#### 2.3.2. Traffic Accessibility Measurement

Traffic accessibility refers to the ability to overcome spatial separation [31]. Generally speaking, this ability is two-fold. It can reach a wider range of space in a shorter time [32]. The improvement of accessibility in space is accompanied by the improvement of accessibility in time. However, when the accessibility in space reaches a ceiling, the accessibility in time can also be improved through technological innovation, such as the transformation from the common railway to the high-speed railway. Considering that the timespan of this study is relatively long, and the early data needed to calculate the temporal accessibility are not easy to obtain, the study mainly discusses the comparative advantages of traffic conditions in the MRYRUA. Thus, spatial accessibility was selected as the basic theory. This study refers to the traffic accessibility calculation method and grading method for the evaluation index developed by Zeng et al. (2018) to calculate the traffic accessibility based on traffic density (the guarantee degree of a location’s own traffic facilities) [26] and traffic convenience (the convenience degree of communication with the outside world). Specifically, traffic density can be represented by road density, railway density, and navigable river density, while convenience is calculated by five indicators (e.g., distance from road, distance from railway, distance from city center, distance from airport, and distance from wharf).

As for the weight of indicators, railways, highways, and airports are functionally more responsible for external exchanges, because there are few pick-up points in a district or county (usually 0–2). Relatively speaking, national highways and provincial roads have played a greater role in internal circulation. In addition, even railways, highways, and airports have different functions. For example, when people are moving within urban agglomerations, high-speed rail is often the best choice; when people need to travel long distances, airplanes are more suitable. In fact, it is difficult for both researchers and travelers to distinguish which function is more important at this stage because of the different advantages. Therefore, this research refers to the results of Zeng et al. (2018) and Feng et al. (2009) and uses an equal weight treatment for different transportation systems [26,27]. Thus, the specific equations are as follows:

(3) $$TA=\frac{1}{2}\times \left(TD+TC\right)$$

(4) $$TD=\frac{1}{3}\times \left(RD+RailD+NRD\right)$$

(5) $$TC=\frac{1}{5}\times \left(DR+DRail+DCC+DA+DW\right)$$

where *TA* stands for traffic accessibility; *TD* and *TC* stand for traffic density index and traffic convenience index, respectively; *RD*, *RailD*, and *NRD* are road density, railway density, and navigable river density, respectively; *DR*, *DRail*, *DCC*, *DA*, and *DW* are realized through the nearest neighbor function of ArcGIS and represent the distance from road, distance from railway, distance from city center, distance from airport, and distance from wharf, respectively.

#### 2.3.3. Spatial Autocorrelation Analysis

The core content of the exploratory spatial data analysis method is to explore whether the attribute values are expressed as spatial aggregation or spatial outliers. Generally, the spatial correlation mode (convergence or heterogeneity) is measured and tested by global Moran′s *I* index and local Moran′s *I* index (LISA) [33]. Global spatial autocorrelation is used to verify the clustering regime of a certain attribute value in the whole regional spatial distribution [34]. The equation is as follows:

(6) $$I=\frac{n}{\sum_{i=1}^{n}\sum_{j=1}^{n}W_{ij}}\times \frac{\sum_{i=1}^{n}\sum_{j=1}^{n}W_{ij}\left(x_{i}-\bar{x}\right)\left(x_{j}-\bar{x}\right)}{\sum_{i=1}^{n}{\left(x_{i}-\bar{x}\right)}^{2}}$$

where *x_i_*, *x_j_* are the observed values; $\bar{x}$ is the average value of the observed attributes; *W_ij_* is the spatial weight adjacency matrix of the spatial element of *i* and *j* (*i*, *j* = 1, 2, 3,…n). The global Moran′s *I* value generally adopts the range between −1 and 1. If it is greater than 0, it indicates that there is a positive spatial autocorrelation; if it is less than 0, it indicates that there is a negative spatial autocorrelation; if its value approaches 0, it indicates random distribution.

Based on Anselin [33], this study adopted the bivariate spatial autocorrelation to explore the spatial relationship between urbanization and traffic accessibility. The Moran′s *I* value produced by bivariate spatial autocorrelation analysis was used to evaluate the correlation degree between one position variable value and other variables with the weighted average value of all adjacent positions [35]. The equation is as follows:

(7) $$I_{kl}^{i}=z_{k}^{i}\sum_{j=1}^{n}\omega_{ij}z_{l}^{j}$$

where *w_ij_* is the spatial connection matrix between spatial units *i* and *j*; $z_{k}^{i}=\frac{x_{k}^{i}-{\bar{x}}_{k}}{\lambda_{k}}$, $z_{l}^{i}=\frac{x_{l}^{i}-{\bar{x}}_{l}}{\lambda_{l}}$; $x_{k}^{i}$ is the attribute value k of the *i*th spatial unit; $x_{l}^{i}$ is the attribute value l of the *j*th spatial unit; $\bar{x_{k}}$ and $\bar{x_{l}}$ are the average value of attribute *k* and *l*; *λ_k_* and *λ_l_* are the variances of attribute *k* and *l*. According to the local Moran′s *I* index, high-high (H-H) type in the calculation results stands for high traffic accessibility and high urbanization level; low-low (L-L) type stands for low traffic accessibility and low urbanization level; high-low (H-L) type stands for high traffic accessibility and low urbanization level; low-high (L-H) type stands for low traffic accessibility and high urbanization level.

#### 2.3.4. Spatial Regression Analysis

In previous studies, the spatial dependence between urbanization and other correlating factors was rarely considered [36,37]. In addition, previous studies tended to favor the presumption that urbanization has no relevance and homogeneity, which leads to incomplete and unscientific research results [37]. In this study, the spatial regression model was introduced for the solution of the complex spatial interaction and spatial dependence between urbanization and traffic accessibility [38,39]. First, the correlation between urbanization and traffic accessibility was measured by the ordinary least square (OLS) method on the whole, without considering the spatial influence of adjacent areas. Then, the spatial regression model suitable for cross-section data was adopted, including the spatial lag model (SLM), spatial error model (SEM), and spatial error model with lag dependence (SEMLD), to explore the spatial relationship between urbanization and traffic accessibility [40,41,42]. The OLS model and spatial regression models were all implemented in GeoDa 095i.

## 3. Results

### 3.1. Urbanization Level in the Middle Reaches of the Yangtze River Urban Agglomerations

**Figure 2:**
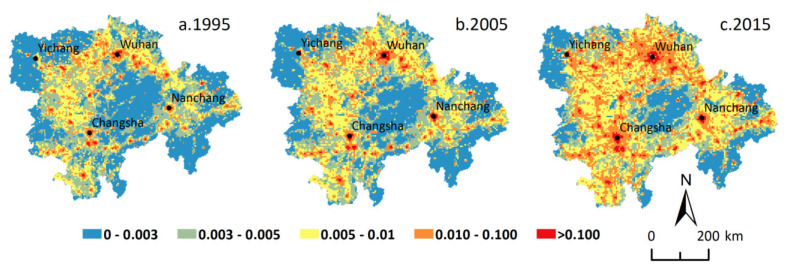
Spatial distribution of urbanization level in the Middle Reaches of the Yangtze River Urban Agglomerations at 5 km grid scale. Note: Reprinted with permission from Chen et al., (2020). Copyright 2020 China University of Geosciences (Wuhan).

**Figure 3:**
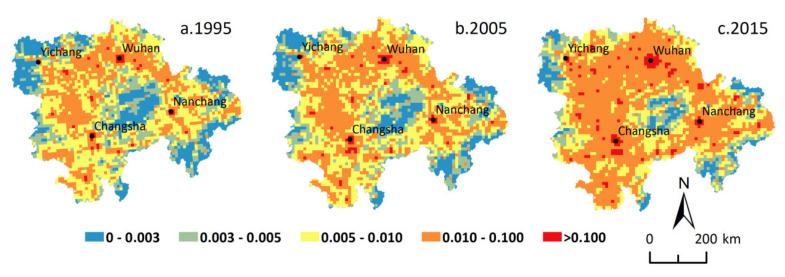
Spatial distribution of urbanization level in the Middle Reaches of the Yangtze River Urban Agglomerations at 10 km grid scale. Note: Reprinted with permission from Chen et al., (2020). Copyright 2020 China University of Geosciences (Wuhan).

In 1995, 2005, and 2015, the urbanization level in the MRYRUA rose sharply at both grid levels. The proportion of grids with increasing urbanization level were 97.27% and 98.01% at a 5 km grid scale during 1995–2005 and 2005–2015, respectively, while the proportion of grids with increasing urbanization level were 97.31% and 98.81% at a 10 km grid scale during the same periods, respectively. The spatial distribution of urbanization levels in the MRYRUA suggested that urbanization in the mountainous areas was generally lower than that of the plain areas, and it was relatively higher in the core areas of these urban agglomerations, the surrounding areas of large cities, and the grids along the main traffic routes, exhibiting the distribution features of ‘point-line-polygon’ (Figure 2 and Figure 3). The urbanization in the MRYRUA descended from the centers of Wuhan, Changsha, Nanchang, and Yichang to the surrounding grids. The linear distribution pattern was found along the traffic network lines in the MRYRUA, and the polygon distribution pattern was conspicuous in core areas of these urban agglomerations. During 1995–2015, the proportion of the urban population in the MRYRUA increased, which gave a boost to the economic development and land expansion. There were obvious spatial spillover effects in the development of urbanization. Not only was the radiation intensity of each core city of the urban agglomeration increasing, but the radiation range was also widening. The spatial pattern of urbanization in the MRYRUA indicated that urbanization was closely related to terrain condition and traffic networks.

### 3.2. Traffic Accessibility in the Middle Reaches of the Yangtze River Urban Agglomerations

**Figure 4:**
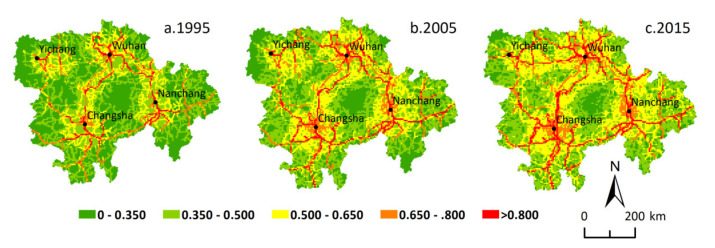
Spatial distribution of traffic accessibility in the Middle Reaches of the Yangtze River Urban Agglomerations at 5 km grid scale.

**Figure 5:**
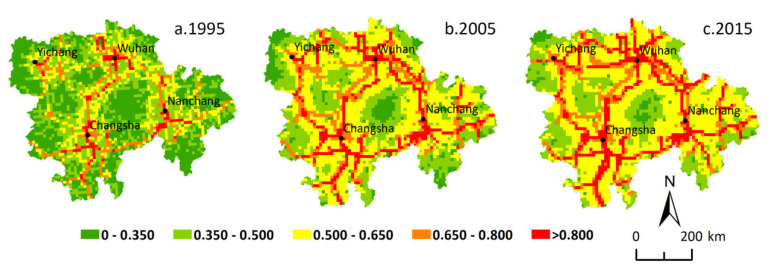
Spatial distribution of traffic accessibility in the Middle Reaches of the Yangtze River Urban Agglomerations at 10 km grid scale.

During the study period, a three-dimensional transportation corridor connecting the domestic large- and medium-sized cities and the neighboring countries came into being in the MRYRUA, with a significant increase in traffic accessibility. In 1995, 2005, and 2015, the average traffic accessibility in the MRYRUA was 0.417, 0.479, and 0.515 at the 5 km grid scale, and 0.432, 0.552, and 0.582 at the 10 km grid scale, respectively. The spatial distribution features of traffic accessibility in the MRYRUA resembled a quadrangle, with Wuhan, Changsha, Nanchang, and Yichang as centers that were radiating towards all directions (Figure 4 and Figure 5). The three-dimensional advantageous traffic networks facilitated the flow of population; this included the railway networks of the Beijing-Guangzhou Line, Beijing-Kowloon Line, Hunan-Guizhou line, and Zhejiang-Jiangxi Line in combination with high-speed roads, national roads, provincial roads, county-level roads, waterways, and other routes of navigation. The core areas of the four major urban agglomerations also manifested an obvious areal distribution, which was self-strengthening and radiated outward over time, thus gradually breaking down the transport barriers between cities and promoting regional integration. The changes in traffic accessibility at different scales during 1995–2005 and 2005–2015 resulted in a proportion of increased grids of 77.42% and 55.48% at a 5 km grid scale and 88.00% and 56.83% at a 10 km grid scale, respectively.

### 3.3. Bivariate Spatial Autocorrelation Analysis between Urbanization Level and Traffic Accessibility

**Figure 6:**
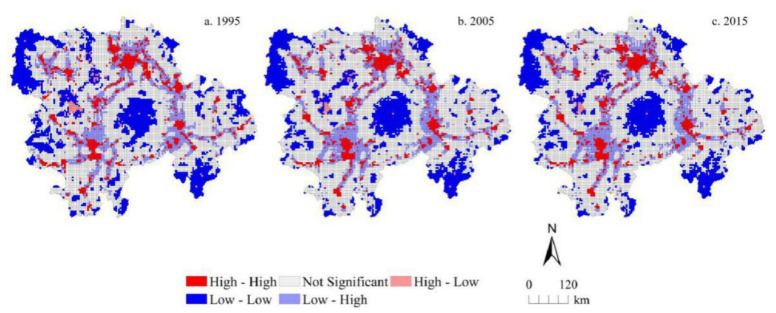
Bivariate LISA cluster maps showing urbanization level and traffic accessibility in the Middle Reaches of the Yangtze River Urban Agglomerations at 5 km gride scale.

**Figure 7:**
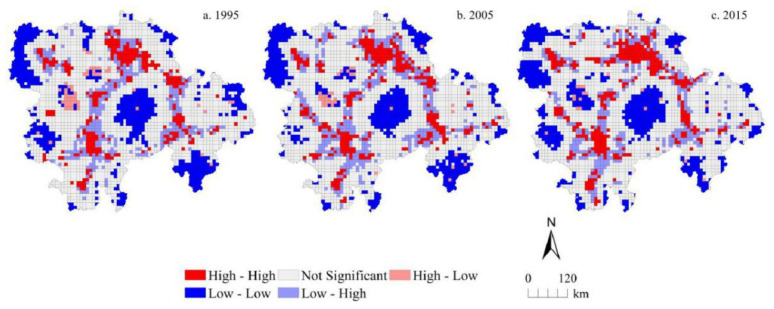
Bivariate LISA cluster maps showing urbanization level and traffic accessibility in the Middle Reaches of the Yangtze River Urban Agglomerations at 10 km gride scale.

Based on GeoDa 095i software, the global bivariate spatial autocorrelation features between urbanization level and traffic accessibility level at different grid scales in 1995, 2005, and 2015 were analyzed. The global Moran′s I index between urbanization level and traffic accessibility level in the MRYRUA was 0.249, 0.260, and 0.303 at a 5 km grid scale and 0.281, 0.277, and 0.310 at a 10 km grid scale for 1995, 2005, and 2015, respectively. We found that the global Moran′s I index at the 10 km grid scale was obviously higher than that at the 5 km grid scale. The results of the global spatial correlation index between traffic accessibility and urbanization level at 5 km and 10 km grid scales in 1995, 2005, and 2015 were all positive and passed the 0.001 significance test. This showed that there was a significant spatial positive correlation between traffic accessibility and urbanization level during the study period. This also proved that strengthening traffic construction can effectively promote the improvement of urbanization level. Some similar spatial features can be found by comparing the LISA aggregation graphs in the bivariate local spatial autocorrelation results (Figure 6 and Figure 7) between traffic accessibility and urbanization level at 5 km and 10 km grid scales. In the years 1995, 2005, and 2015, H-H type (high urbanization level and high traffic accessibility), L-L type (low urbanization level and low traffic accessibility), and L-H type (low urbanization level and high traffic accessibility) were the main relationship types between the traffic accessibility and urbanization level in the MRYRUA. Among them, the H-H type was distributed mainly in the plain areas and surrounding areas of main cities as well as in areas along the main traffic network lines. The L-L type was distributed mainly in the Luoxiao mountain and other mountain areas in the west of Hubei, west of Hunan, and south of Jiangxi, which suggests that these areas were low urbanization level and low traffic accessibility type. L-H type was also distributed mainly in the surrounding areas of the H-H type. These grids with high traffic accessibility but low urbanization level may be potential areas for urbanization.

### 3.4. Spatial Dependence of Urbanization Level on Traffic Accessibility

The results of OLS regression analysis further revealed that there was a significant spatial dependence between urbanization and traffic accessibility (Table 1). The adoption of the OLS regression model, without the consideration of spatial dependence, may lead to incomplete and unscientific interpretation of the relationship between urbanization level and traffic accessibility. These results showed that the spatial regression model significantly enhanced the model’s interpretation ability. Therefore, spatial regression models should be applied in this study. Through the comparison of SEM, SLM, and SEMLD, it could be found that the SEMLD model had larger log-likelihood value and smaller Akaike Information Criterion and Schwarz Criterion value, which can better explain the spatial relationship between traffic accessibility and urbanization level (Table 2, Tables S1 and S2). The regression results showed that there was a significant positive association between urbanization level and traffic accessibility in all models, and it was significant at the level of 0.001. For example, the urbanization level in 1995, 2005, and 2015 would increase by 0.002%, 0.001%, and 0.004% for each 1% increase in traffic accessibility at 5 km grid scale and 0.023%, 0.016%, and 0.027% at 10 km grid scale, respectively. It was also found that the regression coefficients of 5 km grid scale were significantly lower than those of 10 km. The spatial lag terms in all models were significant at the 0.001 level, indicating that the urbanization level was not only affected by the traffic accessibility of the individual grids but also by that of the adjacent grid units. On average, a 1% increase in the surrounding units would result in an increase of the individual urbanization level by 1.162%, 1.147%, and 1.123% at 5 km grid scale, while a 1% increase in the surrounding units would result in the increase of the individual urbanization level by 1.142%, 1.173%, and 1.137% at 10 km grid scale, for the years 1995, 2005, and 2015, respectively. The spatial error terms in all models were also significant at the 0.001 level, indicating that urbanization was also affected by other factors.

## 4. Discussion

### 4.1. Urbanization–Traffic Accessibility Nexus in China

Since the reform and opening-up policy, urbanization in China can be divided into four main stages according to development characteristics [1]. In the first stage (1978–1991), the central government blazed the trail of urbanization, advocating strict control over the scale of large cities, rational development in medium-sized cities, and active scale-up in small cities. During this stage, urbanization occurred mainly in the vicinity of existing cities, and the urbanization process was slow. This initiative effectively transferred the rural labor force and brought about a substantial increase in the number of organic towns. In the second stage (1992–2000), with the establishment of a socialist market economic system, relocating urbanization and in situ urbanization coexisted, speeding up the urbanization process. During this period of transition towards a market economy system, the eastern coastal areas developed rapidly, gradually widening the regional gap and rapidly increasing the number of migrant workers. This set in motion the phenomenal population migration across different regions and between urban and rural areas in China. The third stage (2001–2011) consisted mainly of relocating urbanization. The central government stressed that under the guidance of the actual development requirements, the market mechanism should play an important role in the distribution of resources, thus promoting the coordinated development of large, medium, and small towns and encouraging the surplus rural labor force to make a living in cities. All this promoted the accelerated development of urbanization. The fourth stage (from 2012 to the present) has once again involved the coexistence of relocating and in situ urbanization. Because the past problems with the formal type of urbanization or semi-urbanization were on the radar of the central government, the realization of ‘population urbanization’, with slower growth and higher quality as the driver, has been emphasized during this stage.

**Figure 8:**
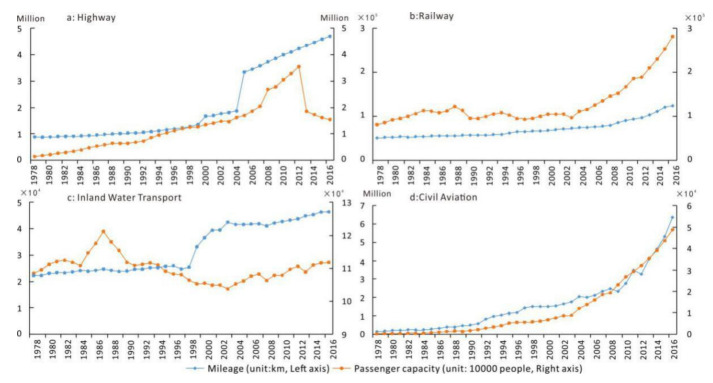
The mileage and passenger capacity of highways, railways, inland water transport, and civil aviation from 1978 to 2016.

Similar to such public services as social security and medical insurance, transportation also falls into the category of the public good provided by the government [43]. The development of transportation corresponds with the characteristics of population migration in each stage (Figure 8). From 1978 to 1990, the trajectory of operation mileage and passenger volume of all kinds of transportation facilities showed that the growth rate of transportation services supply was lower than that of demand; all kinds of transportation operation mileage developed at a very slow speed, while the passenger volume increased at a relatively high speed, especially for highways and railways. From 1990 to the beginning of the 21st century, the passenger volume of highways and civil aviation maintained growth momentum, the railways stabilized, and inland waterway transport reduced significantly. To meet the increasingly prominent transport demand, the government began to accelerate the supply of traffic services. Since the 21st century, China′s infrastructure construction projects have been mushrooming, with land and civil aviation transportation networks entering the high-speed growth stage; inter-modal capacity across different means of transportation in the three-dimensional network has been gradually realized. The corresponding passenger traffic volume of the spatial network system of three dimensions has also shown the same pace of growth. Highway passenger volume plummeted in 2012 and then gradually decreased, which may be closely related to the penetration of high-speed railway in China. The development of transportation bridged the spatial gap between regions. Thanks to expressway and high-speed railway network construction in recent years, the cost of time has been reduced. Inter-regional communication has gradually become a part of daily life, especially in those urban agglomerations with fledged internal transportation systems, such as the Yangtze River Delta Urban Agglomerations.

Previous studies have found that the improvement of the transportation system can effectively promote the development of urbanization in terms of population and developed land; however, the improvement of the transportation network does not necessarily effectively improve the quality of urbanization [44,45]. According to Wiesel′s marginal theory, if the transportation resources cannot fully meet the needs of population migration, economic development, and urban construction in the process of urbanization, further investment in the construction of transportation facilities can effectively promote urbanization. Otherwise, the added investment in transportation would not promote urbanization [46]. The relationship between urbanization and traffic accessibility in the MRYRUA revealed that the improvement of traffic accessibility is still a vital factor to further urbanization, and additional investment in traffic construction was an effective measure to improve urbanization in the study area. For other economies, it would depend on specific situations. When transportation is not an important factor for urbanization, such factors as education level and employment opportunities may be taken into account.

### 4.2. Implications from the Spatial Relationship between Traffic Accessibility and Urbanization

#### 4.2.1. Urban Planning and Development

The level of urbanization was not only related to the regional traffic accessibility but was affected by the transport level of the adjacent areas [47,48]. Rational three-dimensional transport network design can improve the urbanization level of large areas through this spatial spillover effect [45,49,50]. The adjustment or supplement of main and secondary traffic hubs can be determined according to the actual distribution of core areas with high traffic accessibility and high urbanization levels [45]. China is leveraging the urban agglomerations to push urbanization into a critical stage. Urban planning should take urban agglomeration planning as the main manifestation, to be integrated with elements of collaborative urban development design, such as the inflow and outflow of production factors and the easing of administrative restrictions on public resource supply; this can help shape an emerging economy with strong international competitiveness.

Second, the spatial spillover effect of urbanization and transport development also brought about opportunities to the adjacent areas, especially the surrounding areas of core cities [51]. The level of transportation was an important factor affecting the level of urbanization, but it was not the only one. The excessive supply of transportation services or other public services would result in the uneconomical utilization of resources, whereas a moderate and coordinated supply to meet the demand of urbanization development would underpin sustainable development. Without the insufficient supply of supporting public services, development opportunities could be lost. In this light, decision makers should be competent to judge the situation and keep abreast of the development and construction dynamism of neighboring and other core areas. In the area, only by adjusting relevant social welfare supports and improving the local absorption capacity based on experience and foresight can a locality be well-poised to undertake the urbanization spillover brought about by transport development from other places. This can prevent the recurrence of semi-urbanization and deliver ‘population urbanization’.

To conclude, although the spatial patterns of urbanization, data on traffic accessibility, and the results of spatial statistical analysis showed that urbanization and traffic networks were highly coupled spatially, there were still uncoordinated areas. First, the relatively weak transport level and obvious low urbanization level between Changsha-Zhuzhou-Xiangtan Urban Agglomerations and Yichang-Jingzhou-Jingmen Urban Agglomerations indicate that there may be an insufficient supply of traffic services in this area. Second, there were obvious traffic links and low urbanization development levels between Changsha-Zhuzhou-Xiangtan Urban Agglomerations and Wuhan Urban Agglomerations, which came mainly from the large topographic fluctuation in the central region. This may be due to a mismatch of resources or a lack of effectiveness of the traffic links in the area. In the process of urban agglomerations planning, we should pay special attention to such areas, allocate the limited resources reasonably as much as possible, and give full play to their maximum effectiveness.

#### 4.2.2. Sustainable Development

It has been proven that urbanization not only leads to population aggregation, economic growth, and urban land expansion, but also entails some negative external effects, antagonizing the ecological system in such manifestations as the depletion of ecosystem services and the high carbon density of energy consumption [52,53,54,55,56]. In this sense, the development of transportation leads to the improvement of urbanization on the one hand and aggravates the spatial imbalance of the ecosystem on the other hand; this undermines regional sustainable development. The areas with high traffic accessibility and urbanization levels must bear more responsibility for ecological conservation establishing more strict requirements for sustainable development in the transformation of population consumption structure, economic development, and urban growth. They must also be required to offset the negative external effects of urbanization through such mitigation efforts as ecological compensation in the form of free trade under the established emission trading scheme.

The periodic spatiotemporal relationship between traffic accessibility and urbanization has a certain impact on both urban planning and sustainable development. In the irreversible trend of regional integration, urban agglomerations can leverage the transport construction to generate an inflow of labor, capital, and innovative science and technology to enter the class of the developed economies. Relevant stakeholders and public administration departments should effectively grasp the potential of development opportunities and also enshrine the principles of social balance and protection of public resources. The nexus analysis between traffic networks and urbanization levels in this study showed that the optimization and expansion of the traffic network in the MRYRUA should be combined with the development of the urban system in the region. With the improvement of the urbanization levels in the MRYRUA, the optimization and expansion of the corresponding urban and inter-city traffic networks would be carried out accordingly.

### 4.3. Validations and Limitations of This Study

This study measured the impact of traffic accessibility on the level of urbanization development in the MRYRUA using 5 km and 10 km grid scales. To highlight the difference of regional urbanization development levels and traffic accessibility, this study adopted the fishnet tool in ArcGIS10.3 to generate 5 km and 10 km grids as the basic unit of measuring traffic accessibility and urbanization level. This was an improvement in the traditional approach of defaulting to administrative units in evaluation, and involved probing into the scale effect. However, there are also some areas for improvement for further study in this research area. First, the development of a comprehensive measurement model encompassing spatiotemporal accessibility is required. In light of the increasing spatial correlation between traffic accessibility and urbanization level, the trend of diffusion of highly urbanized regions, the convergence impact of traffic accessibility on grid area, and the contribution of temporal accessibility need to be taken into account to further improve the accuracy of this study. Second, China advocates ‘population urbanization’ at present in a bid to realize the transformation from rapid urbanization to high-quality urbanization. Social urbanization is an important objective in the urbanization system and is also an important indicator to evaluate the new-type urbanization levels based on a quality orientation for the future. The timely availability of relevant databases and evaluation systems is the basis for furthering this study. Third, development backgrounds and stages vary between different urban agglomerations and developing economies; this may distort the impact of transport factors on the level of urbanization. In this regard, it is imperative to make suggestions for the integrated urban development plan that reflect empirical research, to improve the applicability of the research. Finally, urbanization and traffic networks are mutually causal. In this study, only the influence of traffic accessibility on urbanization was studied; the influence of urbanization on traffic accessibility was not examined. The relationship between the two needs to be further explored in future studies.

## 5. Conclusions

Based on the three phases of Landsat TM/ETM image interpretation data, 1 km resolution population and GDP raster data, and transport data in the MRYRUA in 1995, 2005, and 2015, this study evaluated the urbanization development level and traffic accessibility in the region. By adopting such techniques as bivariate spatial autocorrelation analysis and a spatial regression analysis model, this study also examined the spatial relationship between urbanization level and traffic accessibility in the MRYRUA in the same period. The main findings are as follows. 

The level of urbanization in the MRYRUA was significantly improved during the study period. The urbanization level in the plain areas, core areas of the urban agglomerations, and areas along the traffic network lines were significantly higher than those of other areas, with the overall spatial layout featuring the pattern of ‘point-line-polygon’. A fully fledged three-dimensional transportation corridor of land, air, and waterway transport networks gradually emerged in the urban agglomerations in the MRYRUA. In general, the traffic networks in the study areas resembled a quadrangle, with Wuhan, Changsha, Nanchang, and Yichang as centers, radiating towards all directions during the study period. The bivariate spatial autocorrelation coefficients between urbanization level and traffic accessibility were both positive and significant during the study period, indicating that there was a significant positive correlation between urbanization level and traffic accessibility. H-H type (high urbanization level and high traffic accessibility), L-L type (low urbanization level and low traffic accessibility), and L-H type (low urbanization level and high traffic accessibility) were the main relationship types between the traffic accessibility and urbanization levels in the MRYRUA. The results of spatial regression analysis illustrated that there was a significant positive correlation between urbanization level and traffic accessibility. The regression coefficient for the 5 km grid scale was significantly lower than that for the 10 km grid scale. In addition, the regression results showed that there was a significant spatial dependence between them; that is, the level of urbanization was not only affected by the traffic accessibility of this grid unit, but also by other adjacent units. The significant spatial spillover effect of traffic accessibility on urbanization indicated that, in the future, the urban planning and traffic system planning processes need to comprehensively consider both the influence of the individual network elements and the adjacent grid units or the farther grid units. In addition, cross-regional collaborative management would improve the rational distribution of urbanization and mobility. A systematic exploration of the nexus between multi-scale urbanization and traffic accessibility can help better understand the interaction mechanism between the two and better serve future planning and policy formulation.

## Figures and Tables

**Table 1:** Regression results of the ordinary least squares (OLS): 1995, 2005, and 2015.

Variable	5 km Scale			10 km Scale		
	1995	2005	2015	1995	2005	2015
TA	0.105 *** (37.968)	0.109 *** (37.960)	0.146 *** (44.001)	0.149 *** (27.894)	0.163 *** (25.499)	0.197 *** (28.247)
Constant	−0.020 *** (−20.603)	−0.026 *** (−22.977)	−0.039 *** (−28.558)	−0.030 *** (−14.888)	−0.050 *** (−16.508)	−0.068 *** (−19.430)
Moran′s I (error)	0.456 ***	0.525 ***	0.562 ***	0.324 ***	0.391 ***	0.431 ***
LM (lag)	9948.601	13,156.942	15,258.694	1167.548	1694.662	2133.661
Robust LM (lag)	228.329	303.252	587.443	27.865	22.715	69.370
LM (error)	9978.904	13,204.583	15,110.331	1245.597	1812.520	2205.706
Robust LM (error)	258.633	350.893	439.081	105.914	140.573	141.415
LM (SARMA)	10,207.233	13,507.835	15,697.775	1273.462	1835.235	2275.076
Log likelihood	22,755.800	22,323.000	21,072.000	5549.570	5060.25	4906.21
AIC	−45,507.600	−44,641.900	−42,140.100	−11,095.100	−10,116.5	−9808.42
SC	−45,492.700	−44,627.000	−42,125.200	−11,083.000	−10,104.3	−9796.23
R-Squared	0.102	0.102	0.133	0.192	0.166	0.196
*N*	12,627	12,627	12,627	3278	3278	3278

**Notes:** TA denotes traffic accessibility. The study uses the queen′s contiguity weight matrix. *** *p* ≤ 0.001. T-stat values are in parentheses. LM = Lagrange multiplier. AIC = akaike information criterion. SC = Schwarz criterion.

**Table 2:** Regression results of the spatial error models with lag dependence: 1995, 2005, and 2015.

Variables	5 km Scale			10 km Scale		
	1995	2005	2015	1995	2005	2015
TA	0.002 (1.511)	0.001 (0.740)	0.004 * (2.211)	0.023 *** (5.631)	0.016 *** (3.730)	0.027 *** (5.416)
Spatial lag term	1.162 *** (176.801)	1.147 *** (195.827)	1.123 *** (202.154)	1.142 *** (59.669)	1.173 *** (69.430)	1.137 *** (72.217)
Spatial error term	−0.481 *** (–25.342)	−0.396 *** (–20.954)	−0.298 *** (–15.924)	−0.385 *** (–10.637)	−0.346 *** (–9.603)	−0.256 *** (–7.193)
constant	−0.003 *** (−6.150)	−0.003 *** (−4.583)	−0.004 *** (−5.580)	−0.011 *** (−8.504)	−0.012 *** (−6.164)	−0.017 *** (−7.421)
Log likelihood	27,759.243	28,512.335	28,105.796	6306.566	6075.334	6095.030
AIC	−55,512.500	−57,018.700	−56,205.600	−12,607.100	−12,144.700	−12,184.100
SC	−55,490.200	−56,996.300	−56,183.300	−12,588.800	−12,126.400	−12,165.800
R-Squared	0.605	0.670	0.719	0.500	0.558	0.614
N	12,627	12,627	12,627	3278	3278	3278

**Notes:** TA denotes traffic accessibility. The study uses the queen′s contiguity weight matrix. *** *p* ≤ 0.001, * *p* ≤ 0.05. T-stat values are in parentheses. LM = Lagrange multiplier. AIC = akaike information criterion. SC = Schwarz criterion.

## Data Availability

The data presented in this study are available on request from the corresponding author.

## Supplementary Materials

The following are available online at https://www.mdpi.com/article/10.3390/ijerph18073828/s1, Table S1: Regression results of the spatial lag model (SLM) and spatial error model (SEM) at 5 km scale, in 1995, 2005, and 2015, Table S2: Regression results of the spatial lag model (SLM) and spatial error model (SEM) at 10 km scale, in 1995, 2005, and 2015.
