# Supplementary material for: The Nexus between Urbanization and Traffic Accessibility in the Middle Reaches of the Yangtze River Urban Agglomerations, China
Source: Int J Environ Res Public Health. 2021 Apr 6;18(7):3828. doi: 10.3390/ijerph18073828 (PMC8038807; doi:10.3390/ijerph18073828)
Supplement: Supplementary file 1 [file ijerph-18-03828-s001.pdf]

Table S1. Regression results of the spatial lag model (SLM) and spatial error model (SEM) at 5 km scale, in 1995, 2005, and 2015.

Variable	1995		2005		2015	
	**SLM**	**SEM**	**SLM**	**SEM**	**SLM**	**SEM**
TA	0.038 ***<br>(18.151)	0.048 ***<br>(16.009)	0.032 ***<br>(16.168)	0.038 ***<br>(13.411)	0.037 ***<br>(17.662)	0.040 ***<br>(13.444)
Spatial lag term	0.823 ***<br>(128.233)		0.869 ***<br>(162.239)		0.891 ***<br>(188.633)	
Spatial error term		0.838 ***<br>(131.581)		0.883 ***<br>(167.885)		0.908 ***<br>(199.534)
Constant	–0.010 ***<br>(–13.980)	–0.001<br>(–0.777)	–0.010 ***<br>(–13.260)	0.000<br>(0.149)	–0.013 ***<br>(–15.106)	0.003<br>(0.996)
Log likelihood	26,202.5	26,155.184	26,849.9	26,801.105	26,452.4	26,378.710
AIC	–52,399	–52,306.4	–53,693.8	–53,598.2	–52,898.8	–52,753.4
SC	–52,376.7	–52,291.5	–53,671.5	–53,583.3	–52,876.4	–52,738.5
R-Squared	0.546	0.546	0.626	0.627	0.689	0.689
N	3278	3278	3278	3278	3278	3278

Notes: TA denotes traffic accessibility. The study uses the queen's contiguity weight matrix. *** $p \leq 0.001$. T-stat values are in parentheses. LM = Lagrange multiplier. AIC = Akaike information criterion. SC = Schwarz criterion.

Table S2. Regression results of the spatial lag model (SLM) and spatial error model (SEM) at 10 km scale, in 1995, 2005, and 2015.

Variable	1995		2005		2015	
	**SLM**	**SEM**	**SLM**	**SEM**	**SLM**	**SEM**
TA	0.081 ***<br>(16.701)	0.111 ***<br>(18.177)	0.076 ***<br>(13.932)	0.113 ***<br>(15.254)	0.084 ***<br>(14.939)	0.114 ***<br>(15.158)
Spatial lag term	0.698 ***<br>(43.599)		0.771 ***<br>(55.821)		0.805 ***<br>(65.147)	
Spatial error term		0.737 ***<br>(45.306)		0.798***<br>(57.779)		0.836 ***<br>(68.631)
Constant	–0.022 ***<br>(–12.882)	–0.017 ***<br>(–5.346)	–0.029 ***<br>(–11.893)	–0.027***<br>(–5.655)	–0.035 ***<br>(–13.216)	–0.027 ***<br>(–4.883)
Log likelihood	6,035.070	6,037.486	5,734.320	5,737.299	5,744.610	5,732.172
AIC	–12,064.100	–12,071.000	–11,462.600	–11470.600	–11,483.200	–11,460.300
SC	–12,045.900	–12,058.800	–11,444.400	–11458.400	–11464.900	–11,448.200
R-Squared	0.451	0.459	0.508	0.515	0.577	0.581
N	12,627	12,627	12,627	12,627	12,627	12,627

Notes: TA denotes traffic accessibility. The study uses the queen's contiguity weight matrix. *** $p \leq 0.001$. T-stat values are in parentheses. LM = Lagrange multiplier. AIC = Akaike information criterion. SC = Schwarz criterion.
